# The protective effect of pericytes on vascular permeability after hemorrhagic shock and their relationship with Cx43

**DOI:** 10.3389/fphys.2022.948541

**Published:** 2022-10-03

**Authors:** Shuangshuang He, Zisen Zhang, Xiaoyong Peng, Yue Wu, Yu Zhu, Li Wang, Henan Zhou, Tao Li, Liangming Liu

**Affiliations:** ^1^ State Key Laboratory of Trauma, Burns and Combined Injury, Shock and Transfusion Research Department, Army Medical Center, Army Medical University, Chongqing, China; ^2^ Department of Pharmacy, Army Medical Center, Army Medical University, Chongqing, China

**Keywords:** PCs, hemorrhagic shock, vascular permeability, lung injury, Cx43

## Abstract

Vascular hyperpermeability is a complication of hemorrhagic shock. Pericytes (PCs) are a group of mural cells surrounded by microvessels that are located on the basolateral side of the endothelium. Previous studies have shown that damage to PCs contributes to the occurrence of many diseases such as diabetic retinopathy and myocardial infarction. Whether PCs can protect the vascular barrier function following hemorrhagic shock and the underlying mechanisms are unknown. A hemorrhagic shock rat model, Cx43 vascular endothelial cell (VEC)-specific knockdown mice, and VECs were used to investigate the role of PCs in vascular barrier function and their relationship with Cx43. The results showed that following hemorrhagic shock, the number of PCs in the microvessels was significantly decreased and was negatively associated with an increase in pulmonary and mesenteric vascular permeability. Exogenous infusion of PCs (10^6^ cells per rat) colonized the microvessels and improved pulmonary and mesenteric vascular barrier function. Upregulation of Cx43 in PCs significantly increased the number of PCs colonizing the pulmonary vessels. In contrast, downregulation of Cx43 expression in PCs or knockout of Cx43 in VECs (Cx43 KO mice) significantly reduced PC colonization in pulmonary vessels *in vivo* and reduced direct contact formation between PCs and VECs *in vitro*. It has been suggested that PCs have an important protective effect on vascular barrier function in pulmonary and peripheral vessels following hemorrhagic shock. Cx43 plays an important role in the colonization of exogenous PCs in the microvessels. This finding provides a potential new shock treatment measure.

## 1 Introduction

Hemorrhagic shock, a serious complication caused by war wounds or trauma, is an important cause of multiple organ dysfunction and is associated with high mortality (Powell et al., 2019; Aussel et al., 2021). Ischemia and hypoxia lead to vascular barrier dysfunction and fluid extravasation, resulting in multiple organ dysfunction (Duan et al., 2017). Searching for effective treatment measures is important for critical illnesses, such as severe shock, trauma, and sepsis.

Pericytes (PCs) are a group of mural cells surrounded by microvessels located on the basolateral side of the endothelium (Sun et al., 2021). Their extensions are radial, connected with multiple vascular endothelial cells (VECs), and entangled in the capillary wall (Daneman et al., 2010). Under physiological conditions, the connection between PCs and VECs plays a vital role in the formation, stability, and permeability of the blood vessels (Stratman et al., 2009; Matsunaga et al., 2021). Previous studies have reported that PCs play an important role in a variety of diseases such as myocardial ischemia and diabetic retinopathy (Chen et al., 2013; Mendel et al., 2013; Fang et al., 2020). However, whether exogenous PC infusion plays an important role in protecting the vascular barrier function following a hemorrhagic shock is unknown.

Previous studies have found that communication between PCs and VECs is both direct and indirect (Bai et al., 2021). Structurally, VECs and PCs share a common basement membrane and gap junction, which allows for communication between PCs and VECs (Geevarghese and Herman, 2014; Liebner et al., 2018). Diverse direct contact structures such as peg-sockets, adhesion plaques, and gap junction-like structures are located at the site of PC–VECs inter-digitations (Armulik et al., 2011; Caporali et al., 2017). The gap junction is a special membrane contact structure formed from two opposing hemi-channels on neighboring cells. Adjacent cells can exchange small molecules through gap junctions, enabling direct communication between cells (Liu et al., 2020). Cx43 is a major protein that modulates the gap junction intercellular communication in VECs (Liao et al., 2001). Previous studies have shown that Cx43 plays an important role in many vascular diseases (De Smet et al., 2021; Ren et al., 2021; Wang et al., 2021). However, whether Cx43 affects the colonization of exogenous PCs in microvessels and then affects vascular hyperpermeability and organ function in hemorrhagic shock remains unknown.

To explore the protective effect of PCs on the vascular barrier and organ function and the relationship between exogenous PC colonization in microvessels and Cx43, the present study used a hemorrhagic shock rat model, VEC-specific Cx43 knockout (KO) mice, and Cx43 knockout VECs. The role of PCs in the protection of the vascular barrier function in hemorrhagic shock and their relationship with Cx43 were observed.

## 2 Materials and methods

### 2.1 Materials

Antibodies against PDGFR-β, CD146, α-SMA, NG-2, and CD31 were purchased from Abcam (United States) and stored at −20°C. Antibodies against ZO-1 and VE-cadherin were purchased from Thermo Fisher Scientific (United States) and stored at −20°C. The pericyte culture medium was purchased from ScienCell (United States). Ringer’s solution (LR) was purchased from Kelun Pharmaceutical (Shanghai, China). Dulbecco’s modified Eagle’s medium-F12 (DMEM F12), DAPI, fetal bovine serum (FBS), and trypsin with EDTA were purchased from HyClone (United States). All other chemicals were purchased from Sigma-Aldrich (St. Louis, MO, United States) unless otherwise specified.

### 2.2 Animal preparation and hemorrhagic shock model

Adult male and female Sprague–Dawley (SD) rats (200–220 g) were purchased from Army Medical University (Shanghai, China). Adult B6.Cg-Tg(Tek-Cre)/Nju mice were purchased from the Nanjing Biomedical Research Institute of Nanjing University (Nanjing, China). Adult floxed-Cx43 mice were purchased from Jackson ImmunoResearch Laboratories (United States). The rats were anesthetized with sodium pentobarbital (45 mg/kg). The hemorrhagic shock model was prepared as previously described (Zhao et al., 2020a). The right femoral artery was catheterized to monitor the mean arterial pressure (MAP) using a mercury sphygmomanometer, and the femoral vein was catheterized for fluid infusion. After catheterization, 500 U/kg of heparinized saline was injected for anticoagulation. The hemorrhagic shock rat model was established by bleeding *via* the femoral artery catheter until the MAP was reduced to 30 mmHg and was maintained at this level for 3 h. Meloxicam (5 mg/kg, SC) was used for postsurgical analgesia in rats and mice. The mice (6–8 weeks old and 20–25 g) model preparation used the same method as that used for the rats. After the terminal studies at the indicated time points, the animals were sacrificed by isoflurane inhalation followed by cervical dislocation. The euthanasia method stated within the study was used for all animals. All animal experiments were approved by the Laboratory Animal Welfare and Ethics Committee of Army Medical University (Approval No. DHEC-2012-069) and conformed to the Guide for the Care and Use of Laboratory Animals (NIH, Publication, 2011).

### 2.3 Isolation, culture, and characterization of pericytes from rats or mice

PCs were isolated from weaning rats or mice as previously described (Liu et al., 2014). Briefly, the weaned rats or mice were anesthetized with sodium pentobarbital (45 mg/kg). The eyeballs were flushed with sterile phosphate-buffered saline (PBS) (pH 7.4) 3–5 times. The retinas were sheared into small pieces and placed in type I collagenase (2.5 g/L, 37°C) for 25 min. The lower filtrate was collected and filtered through a 100-µm mesh filter; the upper filtrate was collected and filtered through a 350-µm mesh filter, and the filtrate was cultured in a PC-specific medium. In addition, the lower filtrate was centrifuged for 8 min, and the precipitate was collected and cultured in a PC-specific medium. Five days later, the cells crawling in the culture flask were PCs, and 3–5 generations of PCs were used for further research. For immunofluorescence staining, cells were seeded on confocal Petri dishes for 24 h and then washed three times with PBS. The cells were fixed in 4% formaldehyde for 10 min, permeabilized with 0.1% Triton X-100 for 5 min, and blocked with 5% bovine serum albumin (BSA) for 30 min at room temperature. They were then incubated with antibodies against NG-2, PDGFR-β, CD146, and α-SMA at −4°C for 12 h. The cells were washed with PBS three times, incubated with fluorescein isothiocyanate (FITC) at room temperature for 1 h, and then observed using a confocal microscope (Leica SP5, Germany).

### 2.4 Cultivation of vascular endothelial cells from pulmonary veins

VECs were obtained from the pulmonary veins of SD rats as described previously (Liu et al., 2016a). Briefly, SD rats were anesthetized, and a thoracotomy was performed using sterile instruments. Pulmonary veins were obtained after the heart was removed. After washing six times with sterile PBS, the veins were sheared to 1 mm × 1 mm pieces, which were attached to the bottom of a cell culture flask (Corning, NY, United States) and cultured in DMEM-F-12, 10% FBS, 1% streptomycin, and 1% penicillin for 72 h. To reduce contamination by fibroblasts, heparin (100 U) and VEGF (Sigma-Aldrich) (10 ng/ml) were added to the culture medium to stimulate pulmonary VEC overgrowth, and differential digestion was used to displace pulmonary VECs from the flask wall prior to fibroblast growth. The 3–5 passages of VECs were used in this study.

### 2.5 Western blotting

Western blot analysis was performed as previously described (Zhao et al., 2020b). Pulmonary veins or VECs were lysed using RIPA lysis buffer, and total proteins were separated by sodium dodecyl-sulfate polyacrylamide gel electrophoresis (SDS-PAGE) and then transferred to polyvinylidene fluoride (PVDF) membranes, which were probed with specific antibodies and analyzed using Odyssey CLx (LI-COR, United States).

### 2.6 Adenovirus production

The Cx43^CA^ and Cx43^WT^ adenoviruses were constructed by cloning mutant Cx43 and wild-type (WT) Cx43 into the vector pAdeno-MCMV-MCS-3Flag-P2A-EGFP. The green fluorescent protein (GFP)-adenovirus, Cx43^WT^ adenovirus control, and Cx43^CA^ adenovirus were purchased from Obio Technology (Shanghai, China).

### 2.7 Transfection of Cx43-down and Cx43-up lentiviruses to vascular endothelial cells

VECs (PCs) were transfected with Cx43-down and Cx43-up lentiviruses as described previously (Zhang et al., 2015). Briefly, VECs (PCs) (10^5^) were inoculated into a culture bottle in DMEM-F-12 containing 10% FBS and cultured for 24 h. The culture medium was then replaced with 1 ml of the transfection system and incubated for 12 h. The transfection system contained 700 μl of culture medium, 100 μl of polybrene solution (0.1 mg/ml), and 200 μl of Cx43-down or Cx43-up lentivirus. The transfection system was replaced with the culture medium, and the transfected VECs (PCs) were cultured for further 48 h.

### 2.8 Treatment of hemorrhagic shock rats or mice with cultured pericytes

PCs (10^6^ cells per rat or mouse) + LR solution [2 × blood loss volume (Zhao et al., 2020a)] were injected into rats or mice through the femoral vein, 3 h after the hemorrhagic shock was induced in PC-treated rats or mice. Rats and mice in the control group were injected with the same dose of LR. The lung and mesenteric microvessels of rats or mice were harvested 12 h after treatment.

### 2.9 Measurement of vascular permeability of the lung with Evans blue/fluorescein isothiocyanate-bovine serum albumin

The rats or mice were anesthetized, and Evans blue (EB) (60 mg/kg) or FITC-BSA (9 mg/kg) was injected through the femoral vein (She et al., 2021). After the animals were sacrificed, the pulmonary vessels were slowly washed with PBS through the heart *in vivo* 2 h after FITC-BSA injection or 30 min after Evans blue injection. The lung tissue was removed and embedded in an optimal cutting temperature compound, and frozen sections (5–10 μm thick) were prepared. The infiltration of FITC-BSA into lung tissue was observed using a confocal microscope (Leica SP5). The water on the surface of the lung tissue was dried, and the lung was weighed. PBS was then added, and the tissue was homogenized in an ice bath. After centrifugation (10 min, 8,000 × *g*, 4°C), the supernatant was removed and centrifuged again (10 min, 16,000 × *g*, 4°C). The optical density (OD) of EB in the supernatant was determined using a multimode microplate reader (BioTek, United States). The ratio of the OD value to the weight of the lung tissue was used to reflect pulmonary vascular permeability.

### 2.10 Hematoxylin-eosin staining of the lung

The rats and mice were anesthetized, and the lung tissue was removed and flushed with PBS. The lung tissue was fixed with formaldehyde, embedded in paraffin, and sectioned for HE staining (Zheng et al., 2020). The pathological sections were observed under a microscope (Leica).

### 2.11 Leakage of fluorescein isothiocyanate-bovine serum albumin in the mesenteric microvessels

The rats or mice were anesthetized with sodium pentobarbital (45 mg/kg) and underwent laparotomy. The ileocecal portion of the mesentery was exposed and placed in the observation plate as described previously (Zheng et al., 2020). The mesentery was moistened with 37°C saline to keep it moist and warm throughout the entire procedure. FITC-BSA (9 mg/kg) was intravenously injected into rats or mice, and the fluorescence intensity of FITC-BSA in mesenteric microvessels was observed at 0, 1, 3, and 6 min by inverted intravital microscopy (Hamamatsu, Japan) (Liu et al., 2016b).

### 2.12 Fluorescein isothiocyanate-bovine serum albumin leakage and transendothelial electrical resistance of vascular endothelial cells

FITC-BSA leakage and transendothelial electrical resistance (TER) of the VECs were measured as previously described (Zhang et al., 2015; She et al., 2021). VECs (10^5^ cells/well) were seeded on the upper inserts of the six-well 0.4-μm transwell culture plate (BD Biosciences, Franklin Lakes, NJ, United States). PCs (10^4^ cells/well) were seeded on the other side of the upper inserts at a ratio of PCs to VECs of 1:10 and transferred to a hypoxia incubator (BugBox, Ruskinn, United Kingdom) (37°C in 1% O_2_/5% CO_2_ air) after the cells had grown to confluence. TER was assessed using a voltohmmeter (World Precision Inc., United States) every 0.5 h. Under anoxic conditions, we placed our hands in the incubator to measure TER without affecting the oxygen concentration in the anoxic incubator. The TER value measured in a non-cell chamber was used as the blank control. Resistivity of VECs = (TER-blank control)/measured TER. The FITC-BSA infiltration rate into VECs was determined as previously described (Liu et al., 2016a). FITC-BSA (10 μg/ml) was added to the upper inserts of the transwell, and the medium (200 μl) in the lower chamber was collected for the measurement of fluorescence intensity at 10, 20, 30, 40, 50, and 60 min using a Synergy HT instrument (BioTek, Winooski, VT, United States). An equal volume of the culture medium was added to the lower chambers after medium collection. The leakage of FITC-BSA (%) = (A_10 min_ + A_20 min_ + A_30 min_ + A_40 min_ + A_50 min_ + A_60 min_)/A total, where A represents the fluorescence intensity.

### 2.13 Vascular endothelial cell-specific Cx43 knockdown mice

B6.Cg-Tg (Tek-Cre)/Nju mice and floxed-Cx43 mice were housed in conventional cages. After multiple generations of backcrossing, heterozygous VEC Cx43 knockdown (Tie2-Cre; Cx43 flox/+) mice were obtained by mating Cre mice with Cx43 flox/+ or cx43 flox/flox genotype mice (Schlaeger et al., 1997). All genotypes were confirmed by PCR analysis. The PCR primer sequences were as follows:

Flox-Cx43: forward 5′-CTT​TGA​CTC​TGA​TTA​CAG​AGC​TTA​A-3';

reverse 5′-GTC​TCA​CTG​TTA​CTT​AAC​AGC​TTG​A-3'.

Cre: forward 5′-GCC​TGC​ATT​ACC​GGT​CGA​TGC-3';

reverse 5′-CAG​GGT​GTT​ATA​AGC​AAT​CCC-3'.

### 2.14 Statistical analysis

Statistical analysis was performed using SPSS 17.0 (SPSS Inc., Chicago, IL, United States). Data are presented as mean ± standard deviation of at least three independent experiments. We used the Shapiro–Wilk test to assess whether the data were normally distributed. An independent samples *t*-test was used for experiments between two groups. One-way analysis of variance (ANOVA) and *post hoc* tests (SNK/LSD) were used to analyze the differences between more than two groups. Statistical significance was set at *p* < 0.05.

## 3 Results

### 3.1 Vascular permeability presented a negative correlation with the amount of pericyte in vessels during hemorrhagic shock

**FIGURE 1:**
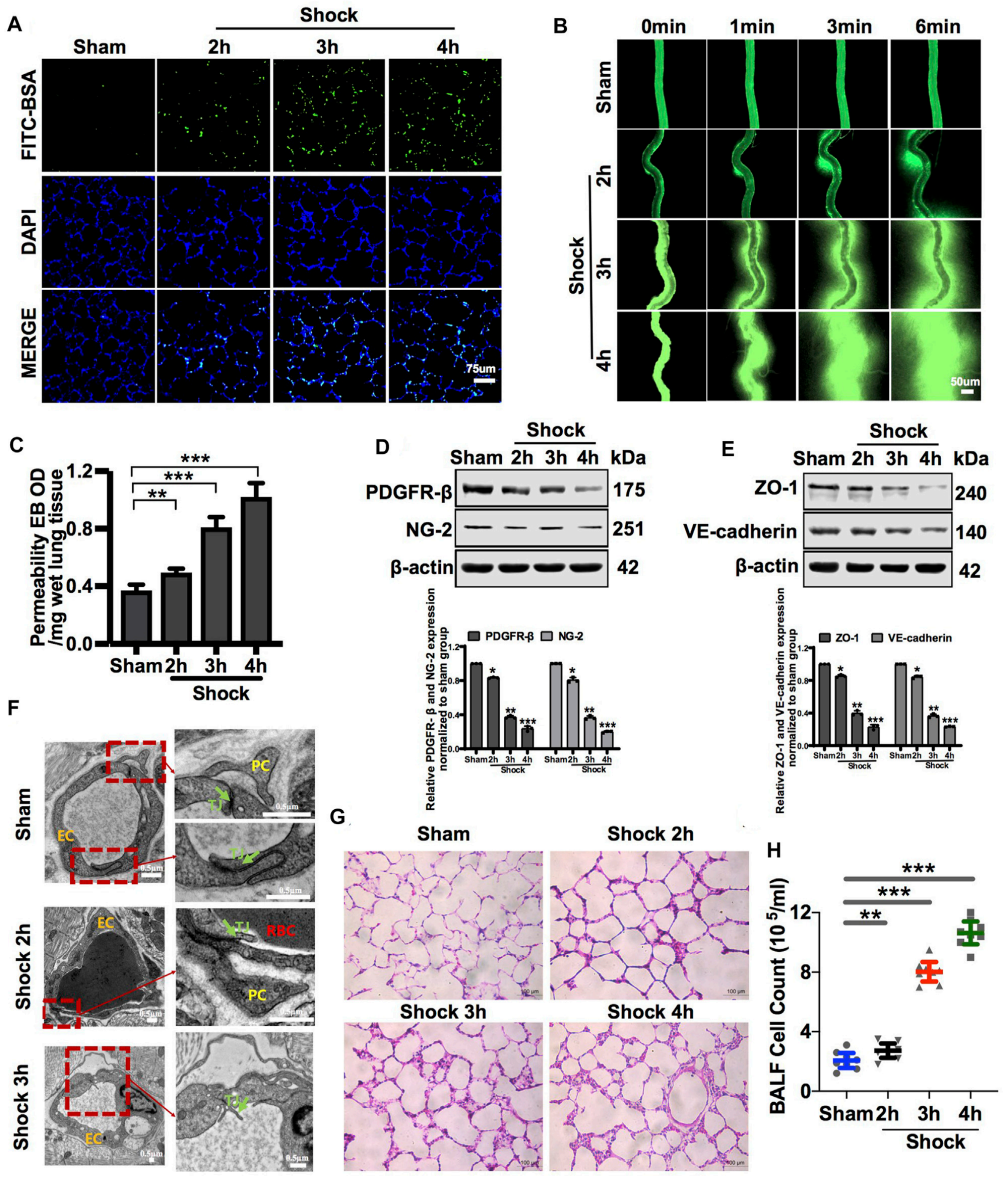
Vascular permeability presented a negative correlation with the amount of PC in vessels during hemorrhagic shock. **(A)** Pulmonary vascular permeability determined by FITC-BSA (*n* = 8). **(B)** Mesenteric microvascular permeability determined by FITC-BSA (*n* = 8 mesenteric microvessels from eight rats). **(C)** Pulmonary vascular permeability determined by EB leakage in lung tissue (*n* = 8). **(D)** Protein expression of PDGFR-β and NG-2 in pulmonary veins after hemorrhagic shock determined by WB (*n* = 12 pulmonary veins from three rats). **(E)** Protein expression of ZO-1 and VE-cadherin in pulmonary veins determined by WB (*n* = 12 pulmonary veins from three rats). **(F)** Structure of PCs and the tight junction structure of VECs observed by TEM (*n* = 32 pulmonary veins from eight rats). **(G)** Infiltration of inflammatory cells in the alveolar space was observed by HE staining (*n* = 8). **(H)** Cell counts in bronchoalveolar lavage fluid (BALF) after hemorrhagic shock (*n* = 8). *p < 0.05, **p < 0.01, and ***p < 0.001 compared with the sham group (one-way ANOVA). RBC, red blood cell; L, lumen; TJ, tight junction.

Following the hemorrhagic shock, FITC-BSA leakage of the pulmonary and mesenteric microvessels was significantly increased in a time-dependent manner during hemorrhagic shock (Figures 1A,B). Meanwhile, EB leakage of pulmonary vessels increased by 34.16%, 120.7%, and 178.85% at 2, 3, and 4 h, respectively, during hemorrhagic shock compared with that of the control group (Figure 1C). Intercellular connections between VECs play a vital role in vascular permeability, as shown in previous research reports (O’Donnell et al., 2014). The ultrastructure of the pulmonary vein was observed by transmission electron microscopy. The results showed that the structure of tight junctions between VECs was complete and continuous in sham rats. In the hemorrhagic shock group, the tight junctions of VECs were damaged, the density of tight junctions was reduced, wide gaps appeared, and shedding was observed (Figure 1F). The adherent junction protein (VE-cadherin) and tight junction protein (ZO-1) are key proteins involved in regulating vascular permeability (Yokota et al., 2021; Zhang et al., 2021). The results showed that following hemorrhagic shock, the expression of junction proteins, such as ZO-1 and VE-cadherin, was significantly decreased (Figure 1E).

The lung is a sentinel organ with multiple organ failures after hemorrhagic shock (Leung et al., 2015; Wu et al., 2021). Vascular hyperpermeability results in the infiltration of inflammatory cells and factors into the alveolar space, leading to lung injury (Wenceslau et al., 2015; Zhang et al., 2015). HE staining showed that the infiltration of inflammatory cells into the alveolar space was significantly increased, and the alveolar walls were thickened (Figure 1G). The number of inflammatory cells in bronchoalveolar lavage fluid (BALF) was increased by 1.32-, 3.89-, and 5.16-fold at 2, 3, and 4 h, respectively, during hemorrhagic shock compared with that of the sham group (Figure 1H).

VECs and PCs share a common basement membrane (Liebner et al., 2018). We found that in sham rats, PCs were located on the basolateral side of the endothelium, which can stabilize the connection of VECs, in line with previous studies (Daneman et al., 2010; Mae et al., 2021). Hemorrhagic shock led to the modification of the PC architecture, followed by PC detachment from VECs (Figure 1F). Following the hemorrhagic shock, the expression of PC markers, such as PDGFR-β and NG-2, in blood vessels was significantly decreased in a time-dependent manner (Figure 1D). Immunofluorescence showed that the expression of PC markers, such as NG-2, in the lung was significantly decreased following hemorrhagic shock (Supplementary Figure S1D). These results suggest that the number of PCs in the vasculature is significantly decreased, whereas vascular permeability is significantly increased during hemorrhagic shock. Loss of PCs plays an important role in vascular hyperpermeability following hemorrhagic shock.

### 3.2 Exogenous pericytes improved the vascular barrier function and alleviated lung injury in hemorrhagic shock rats

**FIGURE 2:**
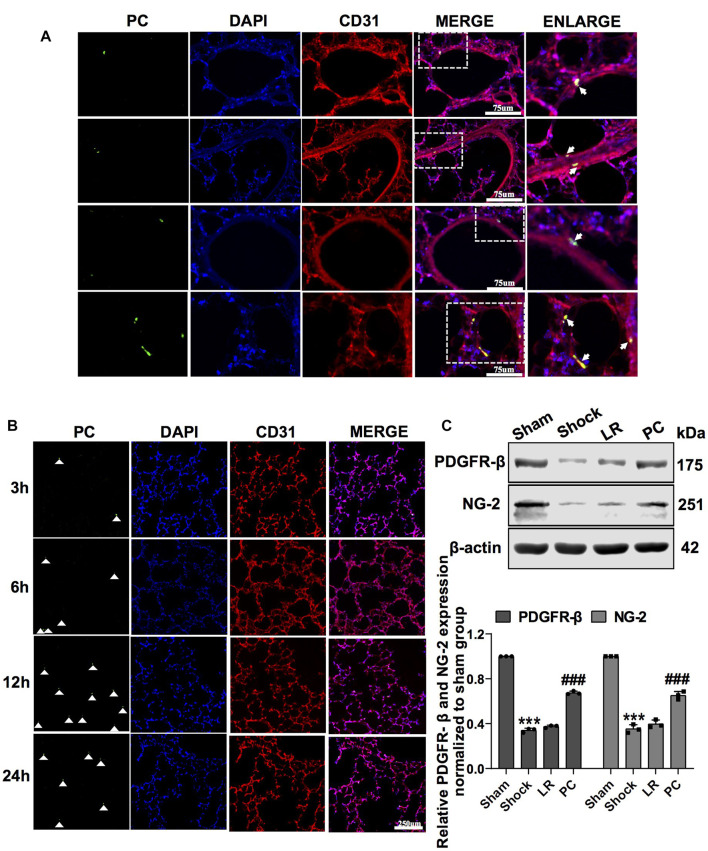
Colonization of exogenous GFP-PCs in pulmonary vessels. **(A)** Location of GFP-PCs colonized in the pulmonary vessels (*n* = 8). **(B)** Colonization of GFP-PCs in the pulmonary vessels at different time points determined by immunofluorescence (*n* = 8). **(C)** Protein expression of PDGFR-β and NG-2 in pulmonary veins determined by WB (*n* = 12 pulmonary veins from three rats). ***p < 0.001 compared with the sham group. ^###^ *p* < 0.001 compared with the LR group (one-way ANOVA).

PCs were transfected with GFP-tagged adenovirus vectors (GFP-PCs) to track injected PCs (Supplementary Figure S1C). To investigate the protective effect and mechanism of action of PCs on vascular barrier function and lung injury during hemorrhagic shock, GFP-PCs were infused into rats *via* the femoral vein. Notably, GFP-PCs were found in the sub-endothelium of the pulmonary vein (white arrow), which indicated that GFP-PCs penetrated the endothelial barrier to the depth of the pulmonary vessels (Figure 2A). We found that exogenous GFP-PCs colonized the pulmonary vessels, and most were colonized 12 h after injection (Figure 2B). WB showed that in GFP-PC-treated rats, the expression of PDGFR-β and NG-2 in pulmonary vessels was significantly increased, whereas no significant change was observed in LR-treated hemorrhagic shock rats (Figure 2C). These results suggest that exogenous GFP-PCs may colonize the pulmonary vessels and significantly supplement the number of PCs in the pulmonary vessels.

Exogenous PCs (10^6^ cells per rat) were transplanted into rats *via* the femoral vein, and vascular permeability and lung injury were observed 12 h after infusion. Immunofluorescence staining was used to identify PCs. The results showed that PC markers such as PDGFR-β, NG-2, CD146, and α-SMA were positive, and CD31 was negative (Supplementary Figure S1A). The characteristics of the PCs were in accordance with a previous report (Zhao et al., 2020a).

**FIGURE 3:**
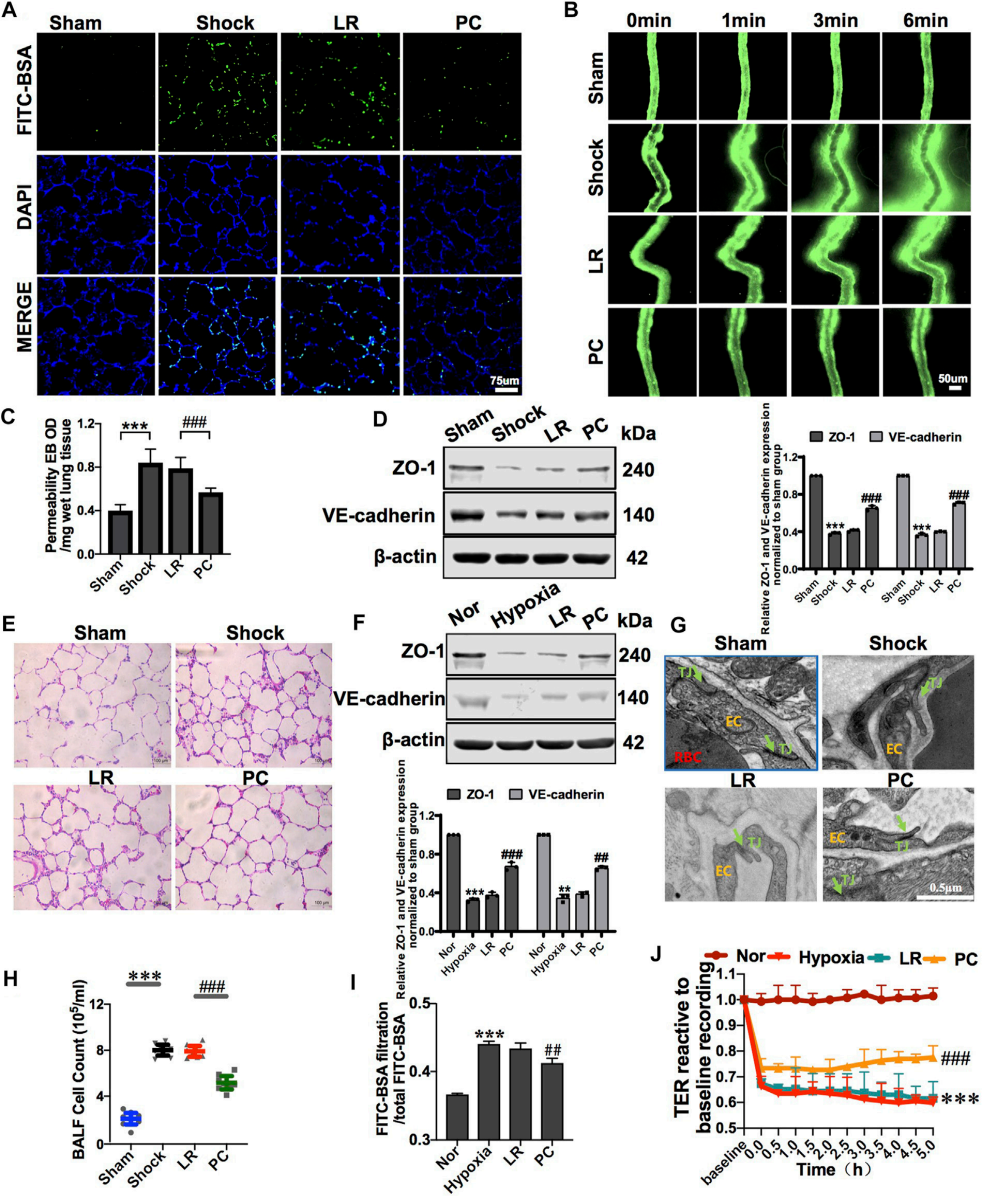
Exogenous PC infusion improved vascular barrier function and alleviated lung injury in hemorrhagic shock. **(A)** Effect of exogenous PCs on pulmonary vascular permeability determined by FITC-BSA (*n* = 8). **(B)** Effect of exogenous PCs on mesenteric microvascular permeability determined by FITC-BSA (*n* = 8 mesenteric microvessels from eight rats). **(C)** Effect of exogenous PCs on pulmonary vascular permeability determined by the EB leakage in lung tissue (*n* = 8). **(D)** Effect of exogenous PCs on the expression of ZO-1 and VE-cadherin in pulmonary veins determined by WB (*n* = 12 pulmonary veins from three rats). **(E)** Effect of exogenous PCs on the infiltration of inflammatory cells in the alveolar space after hemorrhagic shock observed by HE staining (*n* = 8). **(F)** Effect of PCs on the protein expression of ZO-1 and VE-cadherin in VECs after hypoxia determined by WB (*n* = 3). **(G)** Effect of exogenous PCs on tight junction structure of VECs observed by TEM (*n* = 32 pulmonary veins from eight rats). **(H)** Effect of exogenous PCs on the amount of cell in BALF after hemorrhagic shock (*n* = 8 rats/group). **(I)** Effect of PCs on FITC-BSA leakage of VECs after hypoxia (*n* = 3). **(J)** Effect of PCs on the TER of VECs after hypoxia (*n* = 3). **p < 0.001 and ***p < 0.001 compared with the sham group (one-way ANOVA). ##*p* < 0.001 and ###*p* < 0.001 compared with the LR group (one-way ANOVA).

PC transplantation significantly improved vascular barrier function. The leakage of FITC-BSA or EB in pulmonary vessels and mesenteric microvessels was significantly decreased (Figures 3A–C), the structure of the tight junctions was significantly improved (Figure 3G), and the expression of junction proteins, such as ZO-1 and VE-cadherin, was upregulated (Figure 3D), while there were no significant changes in these parameters in the LR-treated group. Meanwhile, PC transplantation significantly reduced the infiltration of inflammatory cells into the alveolar space and BALF (Figures 3E,H). These results suggest that exogenous PC infusion may improve vascular barrier function and alleviate lung injury during hemorrhagic shock, which may be related to the improvement of intercellular junctions of VECs, including the improvement of structure and related protein expression.

To further investigate the effect of PCs on the vascular endothelial barrier function *in vitro*, changes in the permeability of VECs co-cultured with PCs following hypoxia were observed. The results showed that the TER of VECs was significantly decreased, and the leakage of FITC-BSA was significantly increased after hypoxia treatment compared with the control group. PC co-culture improved the barrier function of VECs under hypoxic conditions. In the PC group, TER was significantly increased, and the leakage of FITC-BSA was significantly decreased (Figures 3I,J). The expression of junction proteins, such as ZO-1 and VE-cadherin, significantly decreased after hypoxia. VEC co-cultured with PCs showed upregulated ZO-1 and VE-cadherin expression (Figure 3F). These results further suggest that PCs significantly upregulate the protein expression of intercellular junctions of VECs and improve VEC barrier function after hypoxia.

### 3.3 Pericytes improve vascular barrier function through Cx43

#### 3.3.1 Knockdown and over-expression of Cx43 affected the direct action of pericytes and vascular endothelial cells and pericyte colonization

##### 3.3.1.1 *In vitro*

**FIGURE 4:**
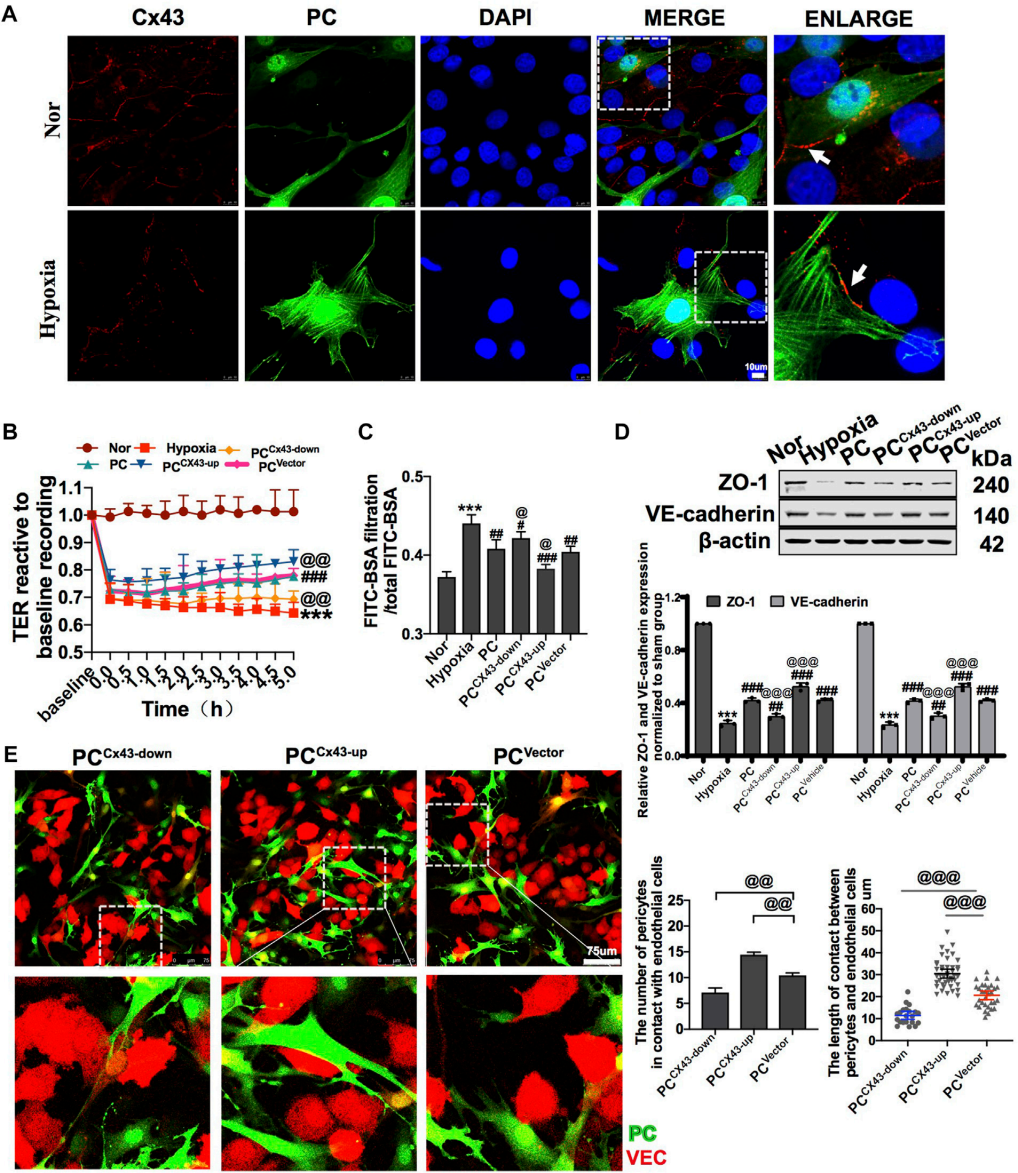
Effect of regulating Cx43 expression in PCs on the action of PCs and VECs and VEC barrier function after hypoxia. **(A)** Expression of Cx43 between PCs and VECs after hypoxia determined by immunofluorescence (*n* = 3). **(B)** Effect of Cx43 up- and downregulation in PCs on the TER of VECs after hypoxia (*n* = 3). **(C)** Effect of Cx43 up- and downregulation in PCs on the infiltration rate of FITC-BSA of VECs after hypoxia (*n* = 3). **(D)** Changes of expression of ZO-1 and VE-cadherin in VECs after treatment determined by WB (*n* = 3). **(E)** Direct contact between PCs and VECs observed using a fluorescence microscope (*n* = 3). ***p < 0.001 compared with the Nor group (one-way ANOVA). ^#^ *p* < 0.01, ^##^ *p* < 0.01, and ^###^ *p* < 0.001 compared with the hypoxia group (one-way ANOVA). ^@^ *p* < 0.01, ^@@^ *p* < 0.01, and ^@@@^ *p* < 0.001 compared with the PC^Vector^ group (one-way ANOVA). PC^Cx43-up^: upregulated Cx43 expression in PCs; PC^Cx43-down^: downregulated Cx43 expression in PCs; PC^Vector^: transfected with adenovirus vectors in PCs.

Diverse direct contact structures such as peg-sockets, adhesion plaques, and gap junction-like structures are located at the site of PC–VEC inter-digitations (Armulik et al., 2011; Caporali et al., 2017). Cx43 is a major protein that modulates gap junctions and intercellular communication (Liao et al., 2001). In *in vitro*, we found that Cx43 expression in PCs and VECs significantly decreased after hypoxia (Figure 4A). We also observed the effect of knockdown and overexpression of Cx43 in PCs on direct contact between PCs and VECs. The results showed that, compared with the PC^Vector^ group (control), the proportion and area of direct contact between PCs and VECs were decreased in the PC^Cx43-down^ group and increased in the PC^Cx43-up^ group (Figure 4E). These results suggest that upregulation of Cx43 expression may promote direct contact, and downregulation of Cx43 may decrease the direct contact of PCs with VECs.

To further investigate the effect of regulation of Cx43 expression in PCs on vascular endothelial permeability after hypoxia *in vitro*, GFP-PCs and VECs were co-cultured. The results showed that compared with the PC^Vector^ group, the TER of VECs was increased and the FITC-BSA leakage of VECs was decreased by 5.57% in the PC^Cx43-up^ group, while the TER of VECs was decreased and that of VECs was increased in the PC^Cx43-down^ group (Figures 4B,C). Moreover, PC^Cx43-up^ upregulated the expression of ZO-1 and VE-cadherin in VECs, as compared with the PC^Vector^ group, and the expression of junction proteins such as ZO-1 and VE-cadherin was significantly increased in the PC^Cx43-up^ group and decreased in the PC^Cx43-down^ group (Figure 4D). These results suggest that upregulation of Cx43 expression in PCs enhances the protective effect of PCs on VECs barrier function, and downregulation of Cx43 expression in PCs weakens the protective effect of PCs on VEC barrier function after hypoxia.

##### 3.3.1.2 *In vivo*

**FIGURE 5:**
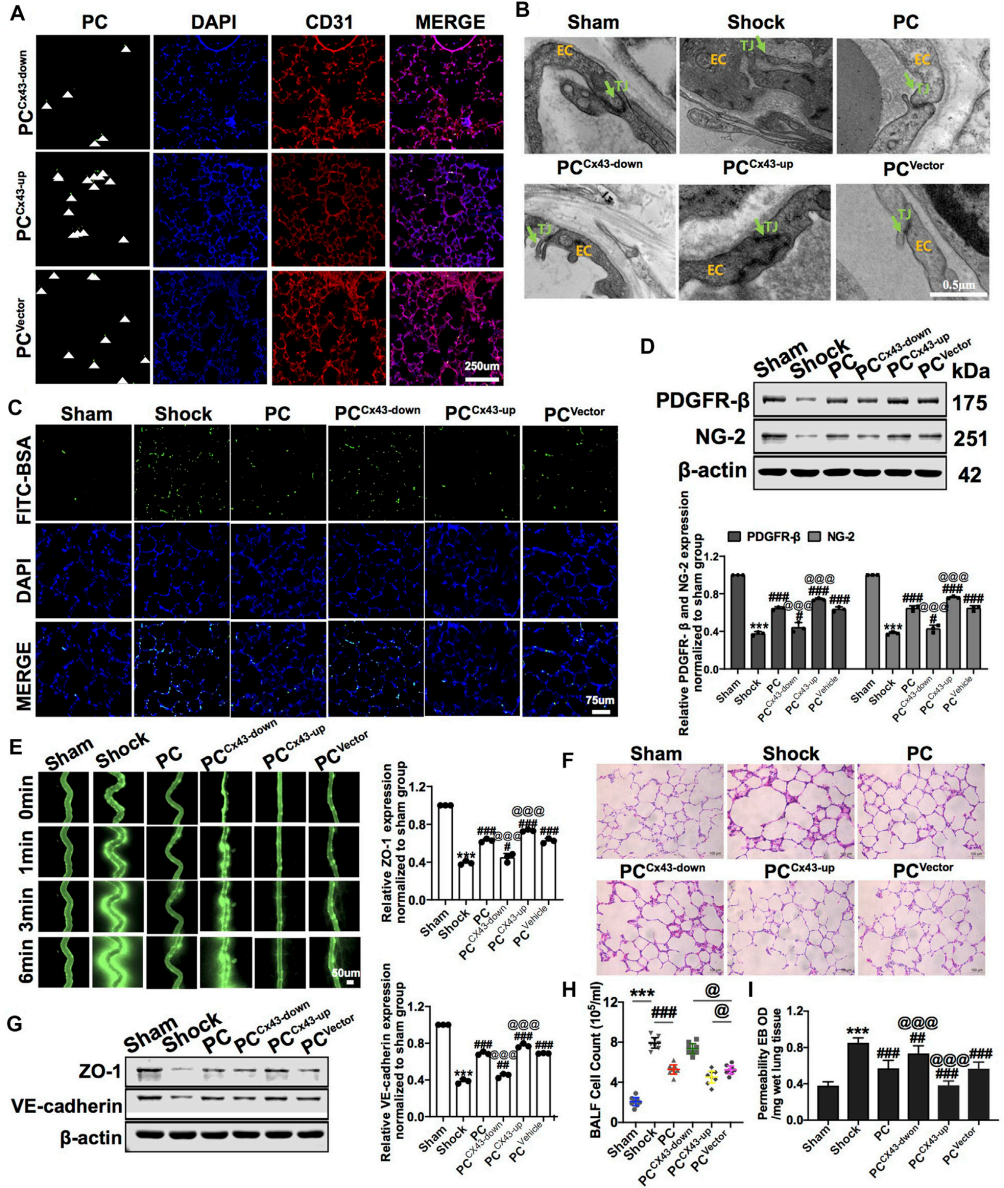
Effect of regulating Cx43 expression in PCs on the colonization of PCs and vascular permeability following hemorrhagic shock. **(A)** Cx43 up- and downregulation in GFP-PCs colonized on the pulmonary veins (*n* = 8). **(B)** Tight junction structure of VECs observed by TEM (*n* = 32 pulmonary veins from eight rats). **(C)** Changes of the pulmonary vascular permeability determined by FITC-BSA (*n* = 8). **(D)** Protein expression of PDGFR-β and NG-2 in pulmonary veins determined by WB (*n* = 12 pulmonary veins from three rats). **(E)** Mesenteric microvessel permeability determined by FITC-BSA (*n* = 8 mesenteric microvessels from eight rats). **(F)** Changes of the infiltration of inflammatory cells in the alveolar space after treatment (*n* = 8). **(G)** Protein expression of ZO-1 and VE-cadherin in pulmonary veins determined by WB (*n* = 12 pulmonary veins from three rats). **(H)** Changes of the amount of cell in BALF after treatment (*n* = 8). **(I)** Changes of the pulmonary vascular permeability determined by EB exudation in lung tissue (*n* = 8). ***p < 0.001 compared with the Nor group (one-way ANOVA). ^#^ *p* < 0.01, ^##^ *p* < 0.01, and ^###^ *p* < 0.001 compared with the shock group (one-way ANOVA). ^@@@^ *p* < 0.001 compared with the PC^Vector^ group (one-way ANOVA).

To explore whether the protective effects were related to Cx43 expression in PCs, we infused exogenous PC^Cx43-up/down^ into hemorrhagic shock rats. The results showed that, compared to PC^Vector^-treated rats, the expression of PDGFR-β and NG-2 was increased, and the colonization of exogenous PC^Cx43-up^ in pulmonary vessels was significantly increased in PC^Cx43-up^-treated rats (Figures 5A,D). The expression of PDGFR-β and NG-2 was decreased, and the colonization of exogenous PC^Cx43-down^ in pulmonary vessels was significantly decreased in PC^Cx43-down^-treated rats compared to PC^Vector^-treated rats (Figures 5A,D).

Compared to PC^Vector^-treated rats, exogenous PC^Cx43-up^ infusion reduced the leakage of FITC-BSA or EB in pulmonary vessels and mesenteric microvessels, whereas exogenous PC^Cx43-down^ infusion increased the leakage of FITC-BSA and EB in pulmonary vessels and mesenteric microvessels (Figures 5C,E,I). Moreover, compared to PC^Vector^-treated rats, the expression of junction proteins, such as ZO-1 and VE-cadherin, was increased in PC^Cx43-up^-treated rats and decreased in PC^Cx43-down^-treated rats (Figure 5G). Compared to PC^Vector^-treated rats, the gaps in tight junctions were narrower, and the density of tight junctions was increased in PC^Cx43-up^-treated rats. In PC^Cx43-down^-treated rats, the gaps in the tight junctions were wider (Figure 5B).

Compared to PC^Vector^-treated rats, exogenous PC^Cx43-up^ infusion reduced the infiltration of inflammatory cells in the alveolar space, downregulated Cx43 expression in PCs, and enhanced lung injury in hemorrhagic shock rats (Figures 5F,H). These results suggest that exogenous PCs have important protective effects on vascular barrier function and lung injury, and Cx43 plays a critical role in this process. Cx43 may affect the colonization of PCs in the microvessels.

#### 3.3.2 Cx43 conditioned knockout in vascular endothelial cells attenuated pericyte colonization and the protective effect

**FIGURE 6:**
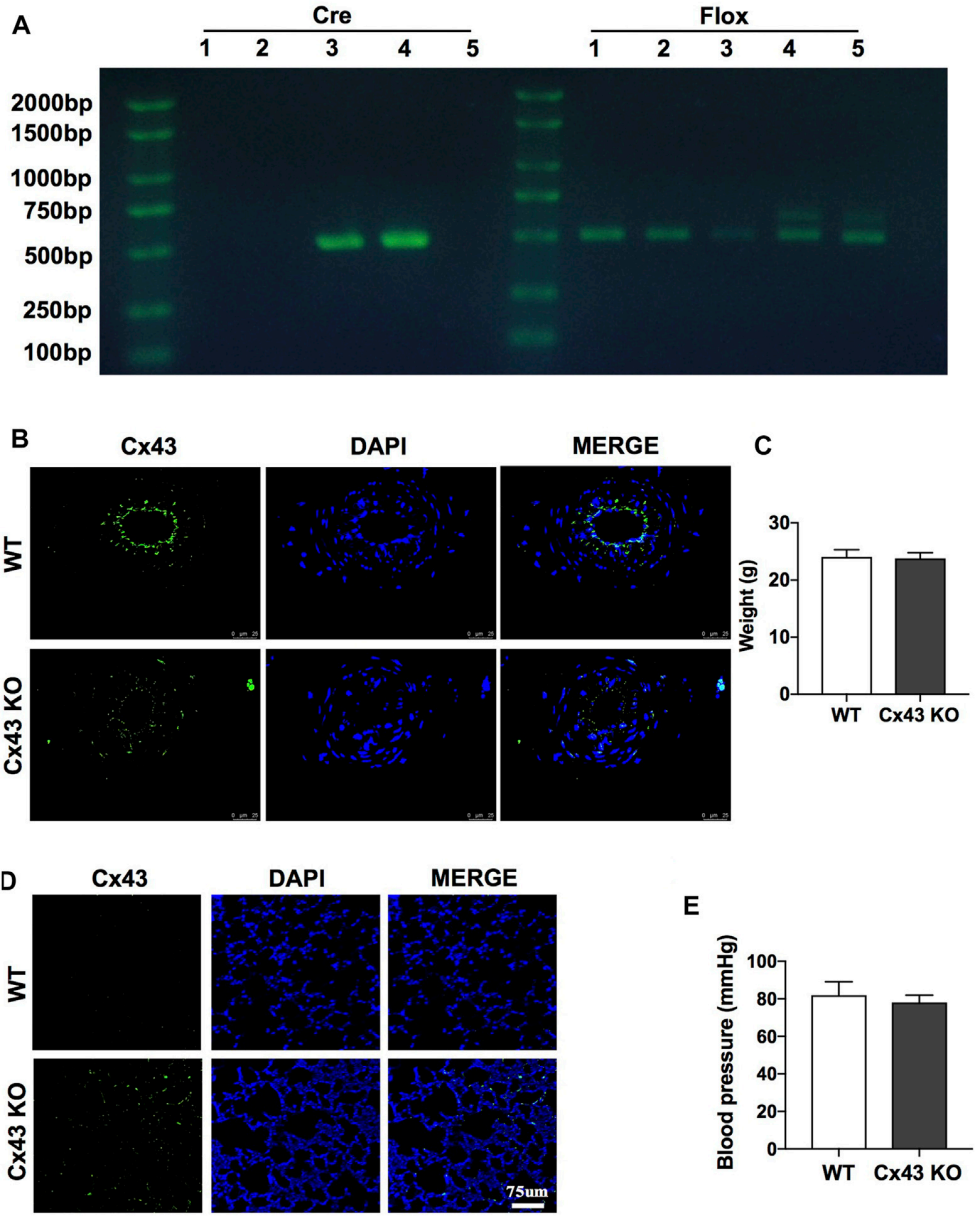
Verification of VEC-specific Cx43 knockdown (VEC Cx43 KO) mice. **(A)** Genomic identification. **(B)** Expression of Cx43 in VECs observed by immunofluorescence (*n* = 8). **(C)** Weight of WT and Cx43 KO mice under normal conditions (*n* = 8). **(D)** Changes of pulmonary vascular permeability in mice (*n* = 8). **(E)** Blood pressure and weight of WT and Cx43 KO mice under normal conditions (*n* = 8).

To further investigate the protective role of Cx43 in vascular permeability and lung injury, VEC-specific Cx43 KO (Tie2-Cre; Cx43^flox/+^) mice (Figure 6A-4th lane) were used. Homozygous VEC-specific Cx43 KO mice (Tie2-Cre; Cx43^flox/flox^) did not survive to maturity, whereas heterozygous VEC Cx43 knockdown mice (Tie2-Cre; Cx43^flox/+^) did survive to maturity. The results showed that the Cx43 expression of vascular endothelium in Cx43 KO mice was significantly decreased (Figure 6B) and the leakage of FITC-BSA in lung vascular was slightly increased (Figure 6D). However, KO of Cx43 in the vascular endothelium did not affect the weight or blood pressure of the mice (Figures 6C,E).

**FIGURE 7:**
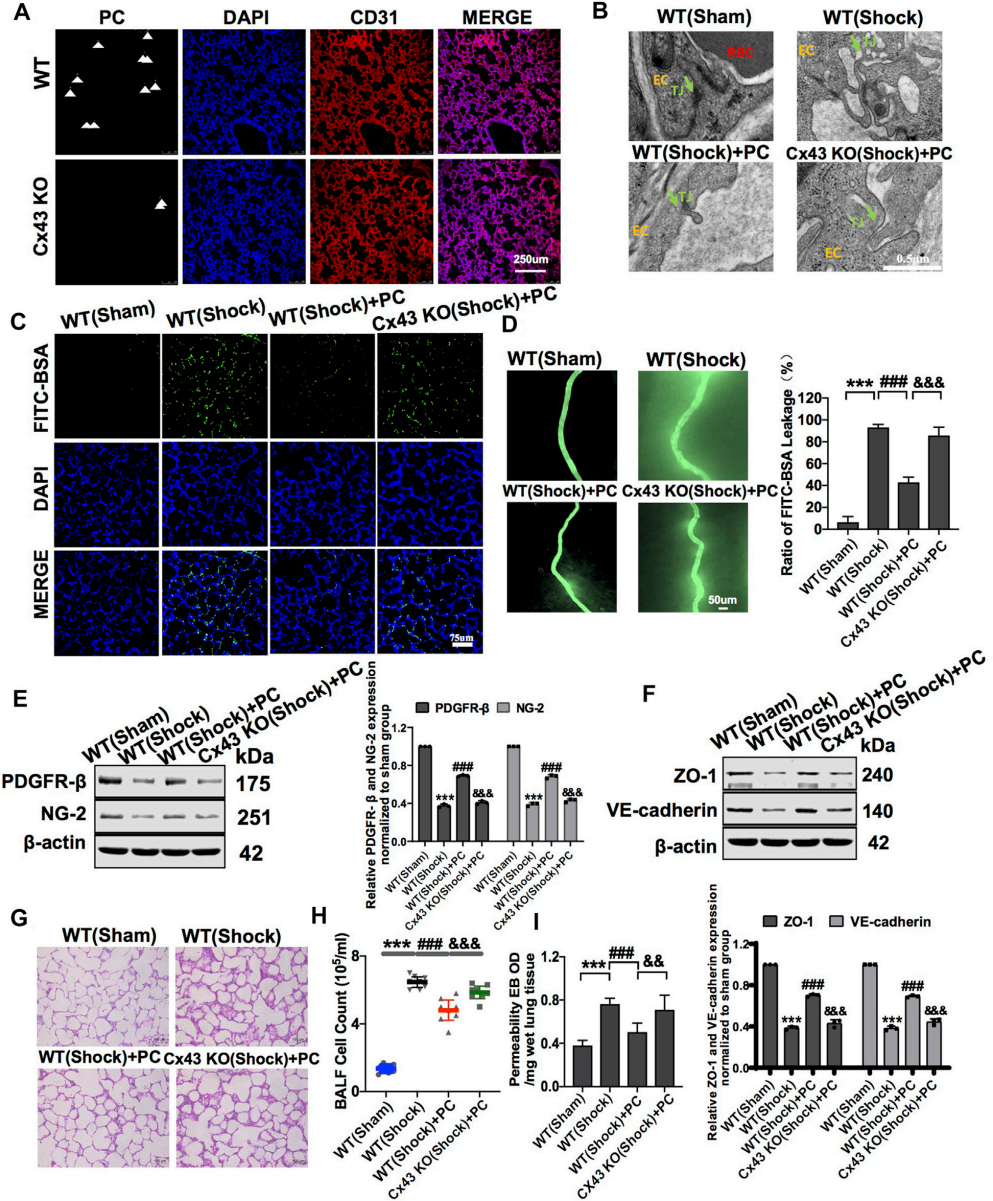
Knockdown of Cx43 in VECs attenuated the protective effect of exogenous PCs on vascular barrier function and lung injury following hemorrhagic shock. **(A)** Colonization of exogenous PCs in pulmonary vessels observed using a fluorescence microscope (*n* = 8). **(B)** Changes of the tight junction structure of VECs observed by TEM (*n* = 8). **(C)** Changes of the pulmonary vascular permeability determined by FITC-BSA (*n* = 8). **(D)** Changes of the mesenteric microvessel permeability determined by FITC-BSA (*n* = 8 mesenteric microvessels from eight mice). **(E)** Protein expression of PDGFR-β and NG-2 in pulmonary veins determined by WB (*n* = 12 pulmonary veins from three mice). **(F)** Protein expression of ZO-1 and VE-cadherin in pulmonary veins determined by WB (*n* = 12 pulmonary veins from three mice). **(G)** Changes of the infiltration of inflammatory cells in the alveolar space observed by HE staining (*n* = 8). **(H)** Changes of the amount of cell in BALF after treatment (*n* = 8). **(I)** Changes of the pulmonary vascular permeability determined by EB exudation in lung tissue (*n* = 8). ***p < 0.001 compared with the WT (sham) group (one-way ANOVA). ^###^ *p* < 0.001 compared with the WT (shock) group (one-way ANOVA). ^&&&^ *p* < 0.001 compared with the WT + PC group (one-way ANOVA).

To further analyze the role of Cx43 in protecting vascular barrier function and lung injury, exogenous PCs were infused into WT and VEC-specific Cx43 KO (Tie2-Cre; Cx43^flox/+^) hemorrhagic shock mice. The results showed that in PC-treated Cx43 KO mice, the expression of PDGFR-β and NG-2 was significantly decreased (Figure 7E), and the colonization of exogenous PCs in the vasculature was significantly decreased compared to that in PC-treated WT mice (Figure 7A).

In Cx43 KO mice, the protective effect of PCs on vascular barrier function was attenuated, and the leakage of EB in pulmonary vessels was increased compared to that in PC-treated WT mice (Figures 7C,D,I). The expression of junction proteins in VECs was significantly decreased, and the integrity of tight junctions of VECs was damaged. In PC-treated WT mice, the expression of junction proteins such as ZO-1 and VE-cadherin was increased, and the tight junctions of VECs were ameliorated as compared to WT control mice (Figure 7F). Cx43 KO in VECs attenuated the protective effect of PCs on tight junction integrity of VECs (Figure 7B).

Meanwhile, the infiltration of inflammatory cells in the alveolar space and the cell count in BALF were increased, and exogenous PC infusion alleviated lung injury in WT hemorrhagic shock. Cx43 KO alleviated the protective effect of PCs on lung injury, the infiltration of inflammatory cells in the alveolar space, and the cell counts in BALF were higher than those in PC-treated WT mice (Figures 7G,H). These results suggest that the knockout of Cx43 in VECs can decrease the colonization of exogenous PCs in pulmonary blood vessels and attenuate the protective effect of PCs on vascular barrier function and lung injury after hemorrhagic shock.

## 4 Discussion

PCs are a group of mural cells around microvessels with multiple functions that participate in the occurrence and development of many diseases such as tumors and neurodegenerative diseases (Winkler et al., 2014; Hosaka et al., 2016; Kovacs-Oller et al., 2020). Previous studies have demonstrated that capillary PCs are rapidly lost after cerebral ischemia in both experimental and human stroke (Fernández-Klett et al., 2013). Our present study found that the number of PCs in the vasculature significantly decreased following hemorrhagic shock. Exogenous PC transplantation can colonize microvessels and effectively increase the expression of intercellular junction-related proteins, ameliorate vascular barrier function, and alleviate organ injury, such as lung injury. The colonization effect of PCs is closely related to Cx43.

PCs have stem cell potential (Mannino et al., 2020). Studies have shown that PCs can differentiate into VECs, vascular smooth cells, myofibroblasts, osteoblasts, and adipocytes by stimulation with growth factors such as TGF-β (Minutti et al., 2019). In recent years, several preclinical and animal studies have explored the role of exogenous PC transplantation in myocardial ischemia (Chen et al., 2013; Fang et al., 2020), chronic liver injury (Lian et al., 2014), and diabetic retinopathy (Mendel et al., 2013), which have been found to be able to treat a variety of diseases. Our present study found that exogenous PC transplantation could colonize the pulmonary and mesenteric vessels, increase the physical coverage of PCs on microvessels, upregulate the expression of intercellular junction proteins, improve vascular barrier function, and alleviate lung injury following hemorrhagic shock.

Previous studies have found that the PCs and VECs communication is exerted by a paracrine mechanism and direct contact action (Bai et al., 2021). The paracrine mechanism is mainly realized through the secretion of bioactive substances or extracellular microvesicles. For example, Shimizu et al. (2012) found that neurotrophic factors derived from PCs increased claudin-5 expression in endothelial cells and improved blood–brain barrier function. Paul et al. found that sphingosine 1-phosphate derived from PCs could upregulate N-cadherin and VE-cadherin expression and downregulate Ang-2 expression in endothelial cells, thereby improving retinal barrier function (McGuire et al., 2011). Our previous study found that PCs could release microvesicles, including Ang-2 and miRNA, to regulate the vasoconstriction response of shock in animals. However, the direct effect of PCs on VECs has not been extensively studied. The present study found that PCs could directly act on VECs to exert protective effects. Exogenous PCs could colonize the vasculature, form a direct connection with VECs, and increase the physical coverage of VECs to exert a protective effect on the vascular barrier function.

Previous studies have demonstrated that there are many direct contact structures between PCs and VECs, such as peg-sockets, adhesion plaques, and gap junction-like structures (Armulik et al., 2011; Caporali et al., 2017). Gap junctions are composed of two hexameric hemi-channels in adjacent cells (Herve et al., 2007; Larranaga-Vera et al., 2021). Cx43 is a major protein that modulates gap junctions and intercellular communication (Liao et al., 2001). Some studies have found that the density of PCs and the expression of Cx43 are reduced in diabetic retinopathy, which could result in vasomotor dysfunction (Muto et al., 2014; Ivanova et al., 2017). A previous study (Bhowmick et al., 2019) has shown that following traumatic brain injury, the expression of N-cadherin, Cx43, and tight junction proteins, such as claudin-5, ZO-1, and JAM-a, was downregulated. Impairment of PC–endothelium crosstalk leads to increased permeability of the blood–brain barrier following traumatic brain injury. The present study found that the expression of Cx43 between PCs and VECs was significantly decreased after hypoxia, which is consistent with previous research. Upregulation of Cx43 promotes direct contact between PCs and VECs *in vitro*. KO of Cx43 in VECs or PCs reduced the colonization of exogenous PCs and attenuated the protective effect of PCs on vascular barrier function and lung injury induced by hemorrhagic shock. It is suggested that Cx43 plays a critical role in the colonization of exogenous PCs in the vasculature.

The present study has some limitations. First, we only investigated the direct effect of PCs on vascular barrier function and whether the paracrine effect of PCs, such as secretion of exosomes, can also have a protective effect on vascular barrier function needs further investigation. Second, in addition Cx43, in the cardiovascular system, there are also other connexins, such as Cx46, Cx45, Cx40, and Cx37, which may also participate in the protective effects of PCs on vascular barrier function. Third, in immunohistochemical experiments and electron microscopy observations, the sample section and staining need to be more elaborate and precise. Finally, we failed to quantify colonized PCs, which is another limitation to the present study.

## 5 Conclusion

PCs have an important protective effect on the vascular barrier function in the pulmonary and peripheral vessels following hemorrhagic shock. Cx43 plays an important role in the colonization of exogenous PCs in the microvessels. This finding provides a potential measure for shock treatment.

## Data Availability

The original contributions presented in the study are included in the article/Supplementary Material; further inquiries can be directed to the corresponding authors.

## Supplementary Material

Supplementary Figure 1 The amount of PC in blood vessels and identification of PCs. **(A)** Identification of PCs by immunofluorescence (n = 3). **(B)** PCs were transfected with GFP-adenovirus (n = 3). **(C)** The protein expression of Cx43 in PCs determined by WB (n = 3). **(D)** The change of the amount of PC in vessels during hemorrhagic shock by immunofluorescence.
