# Supplementary figures and images for: The protective effect of pericytes on vascular permeability after hemorrhagic shock and their relationship with Cx43
Source: Front Physiol. 2022 Oct 3;13:948541. doi: 10.3389/fphys.2022.948541 (PMC9576106; doi:10.3389/fphys.2022.948541)

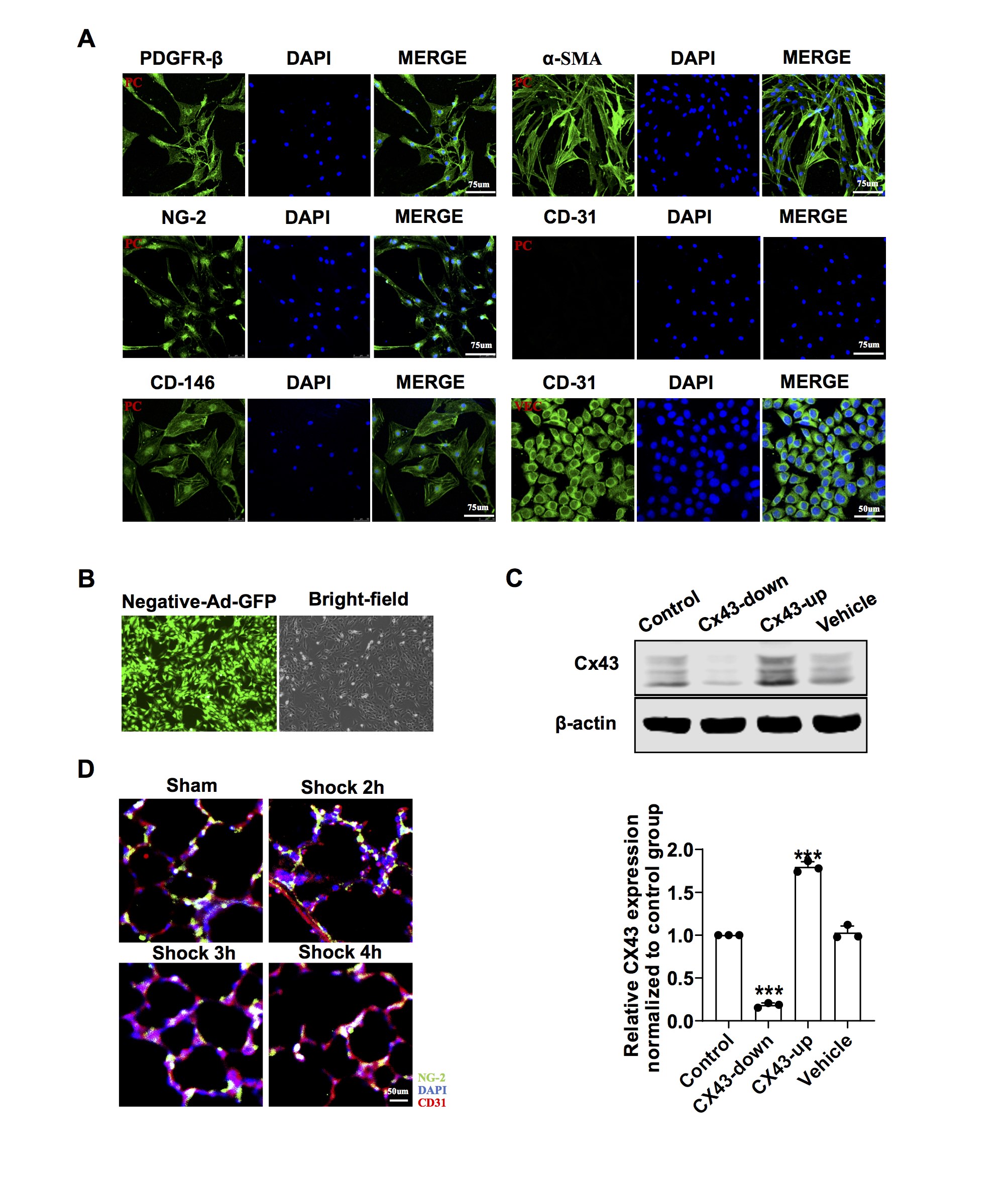

Supplement: Supplementary file 1 [file Image1.jpeg]
